# Clinical Profile of Neurodevelopmental Disorders in Children at a Tertiary Care Center

**DOI:** 10.7759/cureus.67819

**Published:** 2024-08-26

**Authors:** Mridu Bahal, Vineeta Pande, Shradha Salunkhe, Jasleen Dua, Shailaja Mane, Aryan Gupta, Gaurav Kumar, Shivani Kale, Srinija Garlapati, Ruhi Shaligram

**Affiliations:** 1 Pediatrics, Dr. D.Y. Patil Medical College, Hospital and Research Center, Dr. D.Y. Patil Vidyapeeth (Deemed to be University), Pune, IND

**Keywords:** vision disability, intellectual disabilities, autism spectrum disorder (asd), attention deficit hyperactivity disorder (adhd), neurodevelopmental disorders

## Abstract

Background

Neurodevelopmental disorders (NDDs) encompass intricate interactions among genetic, brain, cognitive, emotional, and behavioral processes. These disorders, which are influenced by hereditary and environmental factors, impair personal, social, intellectual, or occupational functioning. Typically emerging early in life, NDDs include conditions such as attention deficit hyperactivity disorder (ADHD), intellectual disability, autism spectrum disorders (ASDs), vision and hearing impairments, motor disorders, and specific learning disabilities. Children from impoverished and low-income neighborhoods are particularly vulnerable. The lack of comprehensive health data and public awareness about these conditions results in limited information regarding the prevalence of neurological illnesses in developing countries. India, with its large and ethnically diverse population, exemplifies this gap.

Methods

It is a prospective study to detect the prevalence and risk factors of neurodevelopmental disorders in children aged six months to nine years at a tertiary care center. Patient details, clinical findings, and relevant history were recorded on a pre-designed pro forma and analyzed statistically.

Results

Among the 1000 children in the study, 91 (9.1%) tested positive for NDDs. Among the 91 children who tested positive for NDD, the highest frequency is in the three to four years age group (17.6%), males were found to be in a higher ratio with 75.82%, with the male: female ratio being 3:1. Among the 91 children with NDD, intellectual disability was the most common disorder (20.9%), followed by ADHD (17.6%) and vision impairment (14.3%). Autism spectrum disorders, including autism and Asperger syndrome, and communication disorders, including stuttering and speech disorders, accounted for 13.2% each. Hearing loss was seen in 9.9% of children and multiple disorders were seen in 8.8% of the children from among 91 children.

Conclusion

Neurodevelopmental disorders are common and often coexist with other conditions. Children from low-income backgrounds are more affected. This study provides valuable insights into the prevalence and characteristics of NDDs in a specific population.

## Introduction

With over 26 million births annually, India has the largest birth cohort globally and has made notable progress in newborn and child survival rates. The country has experienced a significant decline in neonatal, infant, and under-five mortality rates over the past decade. Advancements in the clinical management of preterm infants have led to improved survival rates for premature neonates, including those with both major and minor neurodevelopmental morbidities, which can result in permanent disabilities [1]. Without concurrent interventions, the increased survival of high-risk newborns and children is likely to lead to a higher incidence of neurodevelopmental disorders (NDDs) within the population.

Childhood disability is a major public health concern, linked to low levels of social engagement and development, and poor outcomes in employment, health, and education. Children and adolescents with NDDs face numerous impairments and comorbidities, leading to complex medical, educational, and social support needs. This situation increases the financial and social burden on families, communities, and the nation. Additionally, children with neurodevelopmental impairments are more vulnerable to abuse and neglect compared to their non-disabled peers.

Early identification of developmental issues during follow-up visits allows general practitioners to discuss the need for further neurodevelopmental testing with parents. Screening checklists and questionnaires can facilitate early diagnosis, which may improve outcomes, enable access to specialized care, and prevent severe disability or death [2]. While genetics, chromosomal abnormalities, and metabolic disorders are known contributors to childhood neurological disorders, other significant risk factors include prenatal and post-natal conditions, socio-economic factors, and living conditions in resource-poor countries. There is a lack of comprehensive data from developing nations due to insufficient high-quality health data and public awareness. In India, with its large and diverse population, it is crucial to plan services in a socio-culturally appropriate and cost-effective manner. Collecting epidemiological data on the incidence and distribution of diseases is essential for understanding childhood neurodevelopmental disorders [3].

The COVID-19 pandemic, caused by SARS-CoV-2, has profoundly affected lives worldwide, with children experiencing adverse effects from prolonged lockdowns and physical isolation. This has been particularly detrimental for children with developmental issues, comorbidities, and mental health disorders. The increased screen time and isolation during the pandemic have led to a rise in NDDs [4]. Consequently, we conducted a study at a teaching hospital in Western Maharashtra to determine the prevalence, examine the clinical profile, and identify potential risk factors for neurodevelopmental disorders in children aged six months to nine years.

## Materials and methods

Study design and population

This prospective descriptive study was conducted at the outpatient department of Dr. D.Y. Patil Medical College, Hospital, and Research Center, Pimpri, Pune, involving 1,000 children. Detailed histories were collected for each child attending the hospital, and a series of screening tools were applied to identify potential neurodevelopmental disorders. Children who tested positive on these initial screenings underwent a thorough evaluation and clinical examination. The results from the developmental tools and the diagnosis confirmed by a developmental pediatrician further guided detailed assessments to determine the risk factors and specific types of neurodevelopmental disorder present. Approval was obtained from the institutional ethics committee before the commencement of the study (IESC/PGS/2022/37). Informed written consent was obtained from the parents or guardians of all participating subjects.

Screening and diagnostic tools

Comprehensively assessing the clinical profile of neurodevelopmental disorders in the study population involved various screening and diagnostic tools. For autism spectrum disorder (ASD), the Modified Checklist for Autism in Toddlers (M-CHAT) was used [5]. Attention deficit hyperactivity disorder (ADHD) was evaluated using the INCLEN Diagnostic Tool [6] (International Clinical Epidemiology Network). Visual impairment was assessed through the Visual Acuity Test, and hearing impairment was evaluated using either Pure Tone Audiometry or Brainstem Evoked Response Audiometry (BERA). Intellectual disability (ID) was measured with Intelligence Quotient (IQ) and Development Quotient (DQ) tests. Speech and language disorders were assessed using the Language Evaluation Scale Trivandrum for Children (LEST) [7], while learning disorders were identified with the INCLEN Evaluation Algorithm [8].

Participation criteria

All children between six months and nine years of age whose guardians were willing to participate in the study were included. Children with chronic diseases were excluded from the study.

Sample size

Based on a study [1], which reported the prevalence of neurodevelopmental disorders in children aged two to six years as 5% (95% Confidence Interval) and in the six to nine years age group as 6.5% (95% confidence interval), the required sample size was calculated using Win-Pipe software (SST Systems, Inc., Pleasanton, CA). With an allowable error of 5% at a 95% confidence interval, the calculated sample size was 283. To account for a 10% nonresponse rate, the total sample size was adjusted to 315. However, a study population of 1,000 subjects was chosen to accommodate an acceptable difference of 0.3% for screening.

Data analysis

Data collection was systematically performed and entered into a Microsoft Excel spreadsheet (Microsoft Corporation, Redmond, Washington). Preliminary data analysis was conducted using Microsoft Excel 2024 Version 16.86, where categorical variables were represented through frequencies and percentages. Stata Corp Stata/SE 17.0 (Stata Corp., College Station, TX) was utilized for more advanced statistical analysis. Bivariate tables were created to explore associations between variables, and Pearson’s Chi-square tests of independence were performed to assess statistical significance. A multinomial logistic regression analysis was conducted to identify factors influencing the prevalence of neurodevelopmental disorders among children, providing insights into the impact of various predictors on different neurodevelopmental outcomes. This approach ensured a rigorous and comprehensive data analysis, facilitating a detailed understanding of the relationships and patterns observed. A p-value <0.05 was considered statistically significant.

## Results

Among the 91 children with neurodevelopmental disorders (NDDs), intellectual disability was the most prevalent (20.9%), followed by ADHD (17.6%) and vision impairment (14.3%). Autism spectrum disorders, which encompass autism and Asperger syndrome, and communication disorders, including stuttering and speech disorders, each accounted for 13.2%. Further specifics on the clinical spectrum are provided in Table 1.

**Table 1:** Clinical spectrum of NDD among children. ADHD: attention deficit hyperactivity disorder, ASD: autism spectrum disorder, ID: intellectual disability, NDD: neurodevelopmental disorder.

Disorder	Type	Frequency	Percent	Total (%)
ADHD	Hyperactivity	8	8.8%	16 (17.6%)
	Inattention	3	3.3%	
	Mixed	5	5.5%	
ASD	Autism	10	11.0%	12 (13.2%)
	Asperger syndrome	2	2.2%	
	Pervasive developmental disorder-not otherwise specified (PDD-NOS)	0	0.0%	
Hearing loss	-	9	9.9%	9 (9.9%)
Vision impairment	-	13	14.3%	13 (14.3%)
Intellectual disability	Mild	13	14.3%	19 (20.9%)
	Moderate	4	4.4%	
	Severe	1	1.1%	
	Profound	1	1.1%	
Communication disorder	Stuttering	2	2.2%	12 (13.2%)
	Speech disorder	10	11.0%	
Learning disability	Dysgraphia	0	0.0%	2 (2.2%)
	Dyscalculia	1	1.1%	
	Dyslexia	1	1.1%	
Multiple	ASD with ADHD	5	5.5%	8 (8.8%)
	ASD with ID	1	1.1%	
	ADHD with ID	1	1.1%	
	Hearing loss with vision impairment	1	1.1%	
Total	-	-	-	91 (100.0%)

Consanguinity was found to significantly increase the risk of neurodevelopmental disorders (NDDs) by approximately 50% compared to non-consanguinity (p < 0.05). Males were about 2.2 times more likely to develop NDDs than females (p < 0.05). Birth order and family history of developmental disorders did not significantly influence the risk of NDDs. In contrast, antenatal complications significantly affected the risk. Neither the mode of delivery nor the father's education level significantly impacted the risk. However, a high relative risk ratio (RRR) for the father's education suggested a potential association that may not have reached statistical significance due to a high standard error. Moderate acute malnutrition (MAM) significantly increased the risk of NDDs (p < 0.05), whereas severe acute malnutrition (SAM) showed a high RRR but was not statistically significant (p = 0.061). Logistic regression analysis identified consanguinity (p = 0.043) and male gender (p = 0.021) as significant risk factors for NDDs, emphasizing the role of genetic and demographic factors in these disorders. Further details are provided in Table 2.

**Table 2:** Multivariable logistic regression analysis for risk factors for NDDs. NDD: neurodevelopmental disorders, SAM: severe acute malnutrition, MAM: moderate acute malnutrition, RRR: relative risk reduction.

Risk factors	Reference	RRR	Standard error	p-value	95% CI	
Consanguinity	No consanguinity	-				
Present		0.498	0.172	0.043	0.253	0.979
Gender	Girl	-				
Male		2.219	0.768	0.021	1.126	4.373
Birth order	First					
Second		0.687	0.23	0.261	0.356	1.323
Third and greater		0.725	0.508	0.646	0.183	2.865
Family history of developmental disorders	No	-				
Yes		0.761	0.484	0.668	0.219	2.648
Antenatal complications	No	-				
Yes		0.824	0.433	0.013	0.294	2.31
Mode of delivery	Institutional delivery	-				
Home delivery		0.946	0.553	0.925	0.301	2.972
Father's education	Literate	-				
Illiterate		6.498	8.098	0.133	0.565	74.744
Nutritional assessment	Normal	-				
SAM		6.907	7.124	0.061	0.915	52.154
MAM		7.544	3.99	0	2.675	21.274
Religion	Hindu	-				
Muslim		0.241	0.147	0.02	0.073	0.797
Christian		0.884	1.164	0.925	0.067	11.681
Constant/intercept	-	0.213	0.165	0.046	0.046	0.975

## Discussion

Neurodevelopmental disorders (NDDs) are emerging as a major global health concern, particularly in low- and middle-income countries such as India. In this study, among the 91 children with NDD, intellectual disability (ID) was the most common disorder (20.9%), followed by ADHD (17.6%) and vision impairment (14.3%). Autism spectrum disorders, including autism and Asperger syndrome, as well as communication disorders, such as stuttering and speech disorders, accounted for 13.2%. Hearing loss was observed in 9.9% of children with NDD, while learning disabilities were present in 2.29%. Co-occurring disorders were found in 8.8% of the study participants, with ASD and ADHD constituting 5.5% (see Table 1 for details).

In a district model study conducted in Thiruvananthapuram, Kerala [9], aimed at early detection of childhood disabilities in children under six years old and establishing a referral network for diagnosis verification and early intervention therapy at home, 1,329 children were referred to assessment camps. Of these, 43.1% were found to be normal, 24.98% had speech and language delays, and 22.95% had multiple disabilities. Developmental delay was identified in 49.89% of children, intellectual disability in 16.85%, cerebral palsy in 8.43%, visual impairment in 3.31%, and neuromuscular disorders in 1.35% of the children screened, results from this study were similar to our study.

In a study by Kumar et al. [10], the most common neurological conditions were childhood seizures (69%) and developmental delay (9.7%), with some overlap of conditions. Banoo et al. [11] reported that in Kashmir, seizures, cerebral palsy, and CNS infections were the most frequent neurological disorders. Arora et al. [1] found that in a study of 651 patients in Kolkata, 55.6% were children with autism spectrum disorder (ASD). The most common disorders reported in Indian studies vary considerably, depending on the region, age group studied, and methodology used [12]. In our study, cerebral palsy was excluded due to its high prevalence. Consequently, intellectual disability was the second most common disorder, followed by ADHD.

In a comparative study conducted in the US by Straub et al. [13], the prevalence of neurodevelopmental disorders (NDDs) among children with government or private insurance was observed as follows: ASD (1.6% and 1.3%), ADHD (14.5% and 5.8%), speech or language disorder (8.4% and 4.5%), learning disability (1.2% and 0.6%), developmental coordination disorder (0.9% and 0.7%), behavioral disorder (8.4% and 1.5%), and intellectual disability (0.7% and 0.1%). There was a high overlap of NDDs, particularly in children with ASD and intellectual disability, where more than 70% had one or more additional disorders. The prevalence of certain disorders like ASD and ID appears markedly higher in our study compared to the US study. For instance, ID was reported at 20.9% in India versus 0.7% (government-insured) and 0.1% (private-insured) in the US. Such differences could stem from varying diagnostic criteria, awareness levels, cultural factors influencing reporting, or actual differences in incidence.

In our study, we found that the presence of consanguinity significantly increased the risk of neurodevelopmental disorders (NDDs) by approximately 50% compared to non-consanguineous cases (p < 0.05). This aligns with a study in Saudi Arabia, which found that children from consanguineous marriages were 3.5 times more likely to experience sensorineural hearing loss (SNHL) compared to those from non-consanguineous marriages [14]. Zakzouk [15] conducted two epidemiological surveys in Saudi Arabia, ten years apart, involving 9540 individuals from across the Kingdom and 6421 subjects from Riyadh, revealing consanguinity rates of 45% and 47%, respectively. Additionally, males are about 2.2 times more likely to develop NDDs compared to females, with this result also being statistically significant (p = 0.021). This male predominance aligns with several global studies on ADHD, ASD, intellectual disabilities (IDs), and learning disabilities (LDs) [13,16,17].

Birth order and socio-economic status do not significantly impact the risk of neurodevelopmental disorders (NDDs). Similarly, the presence of developmental disorders in the family does not significantly influence the risk. Antenatal complications and the mode of delivery also do not significantly affect the likelihood of developing NDDs. While the father's education level does not significantly impact the risk, the relative risk ratio is relatively high, suggesting a possible trend that may not be statistically significant due to a large standard error. Moderate acute malnutrition (MAM) significantly increases the risk of NDDs (p < 0.05), whereas severe acute malnutrition (SAM) shows a high relative risk ratio but does not reach statistical significance at the 0.05 level (p = 0.061). A study in Bangladesh found that over 50% of children with neurodevelopmental disorders (NDDs) were malnourished [18]. Similarly, research conducted in Kenya identified malnutrition and/or rickets as risk factors for NDDs [19]. These studies underscore the complex and multifaceted nature of NDDs, with prevalence and types varying significantly based on population, geographic region, and research methodologies used.

Early diagnosis and intervention can significantly alter the developmental trajectory of children with NDDs, highlighting the importance of early screening and access to specialized services. Additionally, both genetic and environmental factors play crucial roles in the etiology of NDDs. Genetic predispositions can be exacerbated by environmental influences such as prenatal exposure to toxins, nutritional deficiencies, and perinatal complications. This interplay between genetics and environment underscores the need for a multifactorial approach in both research and treatment.

The comprehensive examination of NDDs reveals a complex interplay of genetic, environmental, and demographic factors influencing the prevalence and manifestation of these disorders. Intellectual disability and ADHD emerge as the most prevalent, but other conditions like ASD, communication disorders, vision impairment, hearing loss, and learning disabilities also significantly contribute to the spectrum of NDDs. The frequent occurrence of comorbid conditions such as ASD and ADHD further emphasizes the need for holistic and individualized approaches to diagnosis and treatment. Early intervention and access to appropriate healthcare services are crucial in improving outcomes for children with NDDs, underscoring the importance of continued research and comprehensive care strategies.

The study was conducted in a tertiary care hospital, which may limit the generalizability of its findings to other healthcare settings. Additionally, the sample size was relatively small, which can impact the statistical power of the study and the reliability of its conclusions. A limited number of participants may not adequately represent the broader population, leading to potential biases. Further research with larger, more diverse populations across various healthcare settings is necessary to validate and extend the findings of this study.

## Conclusions

The findings emphasize the importance of early diagnosis and interventions, considering the diverse risk factors and prevalence rates associated with NDDs. The results also highlight the necessity of further research into less-understood areas, such as the role of socio-economic factors and specific genetic predispositions, to understand and mitigate the impact of NDDs on affected populations. NDDs significantly impact various domains of a child’s life, including academic achievement, social relationships, and family dynamics. Children with NDDs often require individualized educational plans (IEPs) and specialized support to succeed in school. Socially, they may face difficulties forming and maintaining friendships, leading to isolation and low self-esteem. The family unit is also affected, as parents and siblings must adapt to the unique challenges and demands of caring for a child with an NDD. Support systems, including therapy and community resources, play a vital role in alleviating these pressures and fostering a supportive environment. Understanding the complex nature of these disorders, including their overlapping features and impact on daily life, is essential for clinicians, educators, and families working to support affected children. Through early intervention and tailored support, children with NDDs can significantly improve their developmental trajectory, leading to better outcomes and enhanced quality of life. The ongoing collaboration between healthcare providers, educators, and families remains critical in addressing this issue.

## References

[1] Arora NK, Nair MK, Gulati S (2018). Neurodevelopmental disorders in children aged 2-9 years: population-based burden estimates across five regions in India. PLoS Med.

[2] Johnson S, Miller R (2021). The role of early screening tools in the detection and management of neurodevelopmental disorders. Pediatrics.

[3] Jois RS (2019). Understanding long-term neurodevelopmental outcomes of very and extremely preterm infants: a clinical review. Aust J Gen Pract.

[4] Smith J, Doe A (2022). The impact of COVID-19 pandemic on screen time and its effects on neurodevelopmental disorders in children. J Child Psychol Psychiatry.

[5] Robins DL, Fein DA (2011). Modified checklist for autism in toddlers (M-CHAT) also CHAT. Encyclopedia of Clinical Neuropsychology.

[6] Mukherjee S, Aneja S, Russell PS (2014). INCLEN diagnostic tool for attention deficit hyperactivity disorder (INDT-ADHD): development and validation. Indian Pediatr.

[7] Nair MK, Nair GH, Mini AO, Indulekha S, Letha S, Russell PS (2013). Development and validation of language evaluation scale Trivandrum for children aged 0-3 years--LEST (0-3). Indian Pediatr.

[8] Nair MK, Harikumaran Nair GS, Beena M (2014). CDC Kerala 16: early detection of developmental delay/disability among children below 6 y—a district model. Indian J Pediatr.

[9] Silberberg D, Arora N, Bhutani V, Durkin M, Gulati S (2013). Neuro-developmental disorders in India: an INCLEN study (IN6-2.001). Neurology.

[10] Kumar G, Sharma V, Kumar A (2022). Clinical profile of pediatric neurology disorders: a study from a semi-urban medical college in northwestern India. Cureus.

[11] Banoo N, Wani KA, Hussain M (2022). Clinical profile of neurological disorders in children: a hospital-based experience of a tertiary care centre in Kashmir. Int J Contemp Pediatr.

[12] Rao P, Prasad V (2020). Epidemiology of neurodevelopmental disorders in India: regional variations and methodological challenges. Indian J Pediatr.

[13] Straub L, Bateman BT, Hernandez-Diaz S (2022). Neurodevelopmental disorders among publicly or privately insured children in the United States. JAMA Psychiatry.

[14] Almazroua AM, Alsughayer L, Ababtain R, Al-Shawi Y, Hagr AA (2020). The association between consanguineous marriage and offspring with congenital hearing loss. Ann Saudi Med.

[15] Zakzouk S (2002). Consanguinity and hearing impairment in developing countries: a custom to be discouraged. J Laryngol Otol.

[16] Elbasan B, Duzgun I, Oskay D (2013). Profile of children with neurodevelopmental disabilities who are referred to rehabilitation clinics: a pilot study. Turk Arch Pediatr.

[17] Yang Y, Zhao S, Zhang M (2022). Prevalence of neurodevelopmental disorders among US children and adolescents in 2019 and 2020. Front Psychol.

[18] Khatun R, Bin Siddique MK, Khatun MR, Benzir M, Islam MR, Ahmed S, Muurlink O (2024). Nutritional status of children with neurodevelopmental disorders: a cross-sectional study at a tertiary-level hospital in northern Bangladesh. BMC Nutr.

[19] Segre G, Cargnelutti C, Bersani C (2023). Early detection of neurodevelopmental disorders in African children living in informal settlements in Nairobi. BMJ Paediatr Open.

